# The *UT* family of MHC class I loci unique to non-eutherian mammals has limited polymorphism and tissue specific patterns of expression in the opossum

**DOI:** 10.1186/s12865-016-0181-9

**Published:** 2016-11-08

**Authors:** Katina V. Krasnec, Anthony T. Papenfuss, Robert D. Miller

**Affiliations:** 1 Department of Biology, Center for Evolutionary and Theoretical Immunology, University of New Mexico, Albuquerque, NM 87131 USA; 2 Bioinformatics Division, The Walter and Eliza Hall Institute of Medical Research, 1G Royal Pde, Parkville, 3052 Australia; 3 Department of Medical Biology, University of Melbourne, Melbourne, Australia; 4 Peter MacCallum Cancer Centre, East Melbourne, 3002 Australia; 5 Sir Peter MacCallum Department of Oncology, University of Melbourne, Melbourne, Australia

**Keywords:** MHC, Class I, Genomics, Marsupials

## Abstract

**Background:**

The Major Histocompatibility Complex (MHC) class I family of genes encode for molecules that have well-conserved structures, but have evolved to perform diverse functions. The availability of the gray, short-tailed opossum, *Monodelphis domestica* whole genome sequence has allowed for analysis of MHC class I gene content in this marsupial. Utilization of a novel method to search for MHC related domain structures revealed a previously unknown family of MHC class I-related genes. These genes, named *UT1-17*, are clustered on chromosome 1 in the opossum, unlinked to the MHC region. *UT* genes are only found in marsupial and monotreme genomes, consistent with being ancient in mammals yet lost in eutherian mammals. This study investigates the expression and polymorphism of the *UT* loci in the opossum to gain insight into their possible function.

**Results:**

Of the 17 opossum *UT* genes, most have restricted tissue transcription patterns, with the thymus and skin being the most common sites. Full-length structure of 11 UT transcripts revealed genes varying between five and eight exons, typical for class I family members. There were only two alternative splice variants found. The *UT* genes also have limited polymorphism and little evidence of positive selection. One locus, *UT8,* was chosen for further analysis due to its conservation amongst marsupials and generic characteristics. *UT8* transcription is limited to developing αβ thymocytes, and is absent from mature αβ T cells in peripheral lymphoid tissues.

**Conclusion:**

The overall characteristics and features of *UT* genes including low polymorphism and restricted tissue expression make it likely that the molecules encoded by *UT* genes perform roles other than antigenic peptide presentation.

**Electronic supplementary material:**

The online version of this article (doi:10.1186/s12865-016-0181-9) contains supplementary material, which is available to authorized users.

## Background

The Major Histocompatibility Complex (MHC) class I gene family encodes cell surface proteins that share a unique, well-conserved structure. Typical MHC class I-related molecules are heterodimers of α-chains paired with β2-microglobulin [1]. The α-chain contains three extracellular domains (α1, α2, and α3), along with a transmembrane domain and a cytoplasmic tail [2]. The α1 and α2 domains combine to form a groove comprised of a β-sheet and two α-helices, a structure unique to MHC class I proteins [3]. The α3 domain is an immunoglobulin superfamily domain. So far, genes encoding the MHC class I α-chain family have only been found in the gnathostomes, the jawed vertebrates [4].

In spite of the conserved structure, MHC class I molecules have evolved to perform a diverse set of functions. The initial role for MHC class I proteins uncovered was the presentation of antigenic peptides to cytotoxic, CD8^+^ T cells [5]. Antigenic peptides derived from proteolysis of self or pathogen derived proteins are bound in the groove created by the α1 and α2 domains and “presented” to CD8^+^ T cells by forming a ligand for the T Cell Receptor (TCR) [6]. Because this was the first role discovered for MHC class I molecules it is usually termed their “classical” role. Indeed, peptide presentation is the most ubiquitous role for MHC class I molecules and is likely their ancestral function [7]. MHC class I molecules involved in peptide presentation, such as human HLA-A and -B, are characteristically polymorphic and ubiquitously expressed [8].

Some MHC class I related molecules have evolved to present non-peptide antigens to T cells. CD1, for example, presents glycolipids to T cells [9]. Even more wide ranging roles for MHC class I related molecules have evolved such as the neonatal Fc receptor (FcRn) that binds and transports IgG. FcRn transports IgG across the placenta in some mammals, and in some is expressed in mammary tissue and neonatal gut for transmitting maternal IgG through the milk [10]. Although maintaining the traditional MHC class I domain structure, FcRn is unable to bind peptides. The groove formed by the FcRn α1 and α2 domains is occluded and does play a role in binding IgG [11, 12]. Other examples of divergent MHC class I roles include the HFE molecule that associates with the transferrin receptor, reducing the receptor’s affinity to load transferrin molecules bound with iron, and the murine M10 molecule that binds and serves as a chaperone for vomeronasal organ olfactory receptors [13, 14]. In contrast to MHC class I genes encoding molecules that present peptides, those encoding ‟non-classical” molecules such as mouse M10, FcRn, and CD1 are generally less polymorphic and often have tissue specific patterns of expression [15–17]. Exceptions to this rule include non-classical class I such as MIC and Qa1, which have broader tissue distribution and polymorphism [18]. Comparative genomics has facilitated the discovery of novel, non-classical MHC class I molecules and provided insight into the broad plasticity of this protein structure.

The gray short-tailed opossum, *Monodelphis domestica*, is arguably the most established model marsupial species, and the first to have a sequenced genome [19, 20]. Many of the MHC class I genes in the genome of *M. domestica* have been annotated [21–27]. There are three presumed peptide-presenting MHC class I genes in the opossum *Monodelphis domestica* (*Modo*) *UA1*, *UA3*, and *UA4. ModoUA1* and *UA3* display many traits common to peptide presenting MHC genes, such as ubiquitous expression and high levels of polymorphism*. ModoUA4* has lower levels of polymorphism but remains ubiquitously expressed [23, 27]. Many of the other known opossum MHC class I genes encode molecules likely to have non-classical functions. *ModoUG*, for example, can be expressed in three different alternatively spliced mRNA forms [24]. In these forms, a short cytoplasmic tail has been found that does not have traditional phosphorylation sites. *ModoUJ* and *UM* also have several alternative mRNA isoforms, including one possible *ModoUJ* soluble isoform [21]. A majority of the opossum MHC class I genes have limited polymorphism consistent with possible non-classical roles.

The functional plasticity of the MHC class I structure makes these molecules models to study the evolutionary relationship between structure and function. The availability of whole genome sequences from a variety of gnathostome lineages provides the information needed to discover new MHC class I genes and potentially new roles for these molecules. Recently, a new family of MHC class I loci called *UT* was found, and appears to be restricted to marsupials and monotremes [28]. The phylogenetic distribution is consistent with having emerged early in mammalian evolution and subsequently lost in the eutherian lineage. A more in-depth analysis of these genes revealed that some were transcribed in immune tissues of the opossum, tammar wallaby, brushtail possum and Tasmanian devil. The goal of this study was to further characterize the *UT* genes in a model marsupial, *M. domestica.*

## Results

### Analysis of ModoUT transcription

To investigate transcription of *ModoUT* genes in a variety of tissues, a public transcriptome data from the OpossumBase public database was downloaded and searched. This database contains transcriptome profiles of 19 different tissue types. Since OpossumBase lacked data from the thymus, a good candidate tissue for immune gene expression, a separate thymus transcriptome database generated on the Roche 454 platform was also analyzed.

**Fig. 1:**
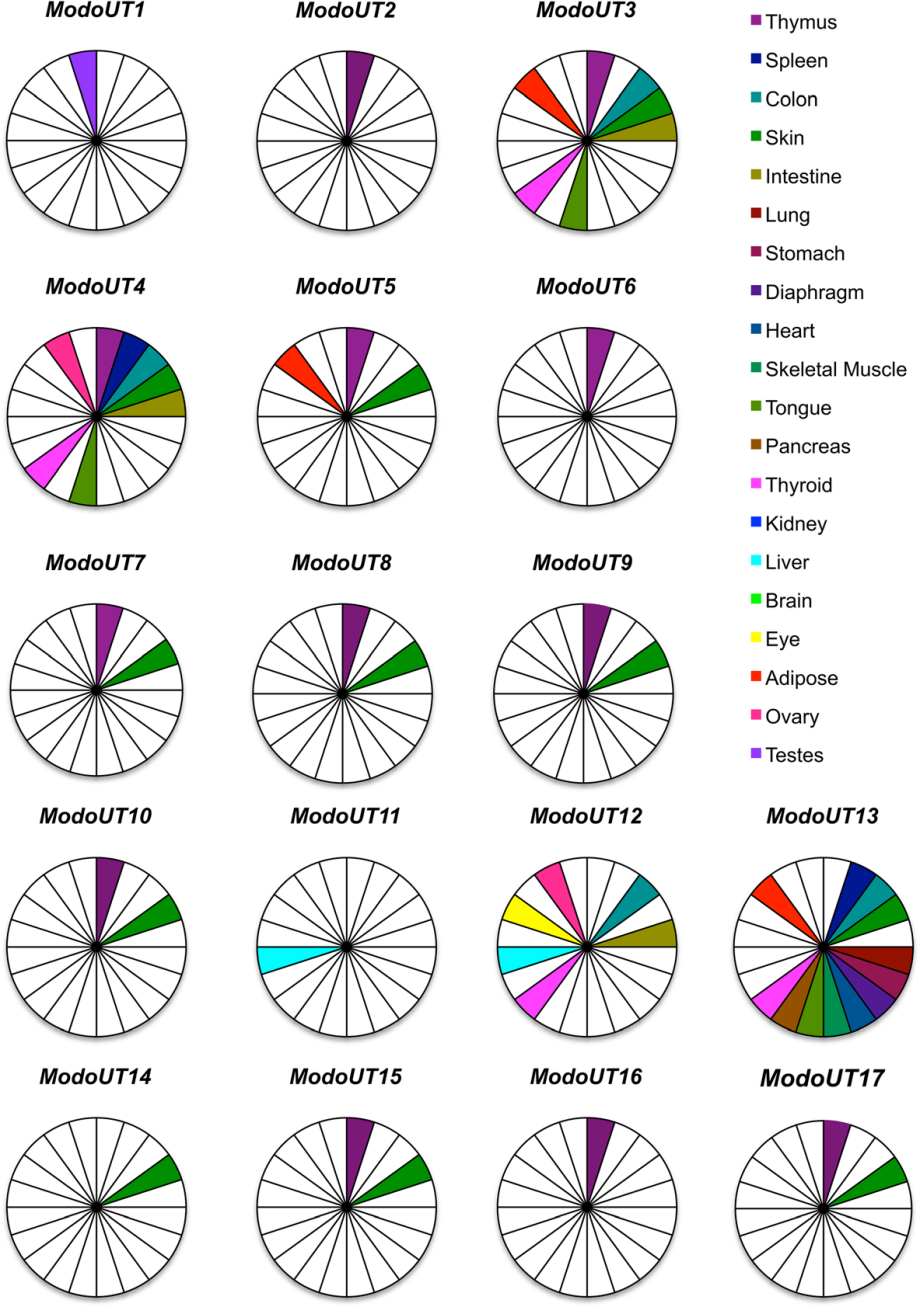
Differential expression of *ModoUT* genes across 20 different tissue types. Filled pie wedges represent the detection of a *ModoUT* transcript in either a Roche 454 dataset or from Illumina sequences found at www.OpossumBase.org with a 99 % sequence identity at 180 or more base pairs. Tissue types examined included lymphoid, epithelial, muscle, endocrine, excretory, reproductive, CNS, and miscellaneous that included the eye and adipose

The initial analyses focused on the complexity of *ModoUT* loci transcribed in each tissue type. Of the 17 *ModoUT* genes, the most common sites for transcription are the thymus and the skin (Fig. 1). Twelve out of 17 *UT* loci are transcribed in the opossum thymus, and eleven out of 17 are transcribed in the skin. For all other adult tissues, the colon and thyroid contained the greatest variety of transcribed *ModoUT* genes with four each. In contrast, the testes, eye, lung, diaphragm, skeletal muscle, pancreas, stomach, and heart each only transcribe a single *ModoUT* locus. Brain and kidney were the only adult tissues investigated that lacked any *ModoUT* transcripts.

Next the breadth of transcription of each *ModoUT* locus among the various tissues was examined. Most of the *ModoUT* genes demonstrated restricted tissue transcription patterns. *ModoUT1, 11,* and *14* were each found transcribed in only a single tissue, the testes, liver, and skin, respectively. Similarly, *ModoUT2, 6,* and *16* were only transcribed in the thymus. In contrast, *ModoUT13* was found most broadly transcribed in 16 different tissues (Fig. 1). All transcript sequences corresponding to *ModoUT7* contained an in-frame stop codon near the beginning of exon 3. Whether *ModoUT7* is a pseudogene or the sequences present in the available databases are transcribed from null alleles is not yet known.

### ModoUT gene organization

**Fig. 2:**
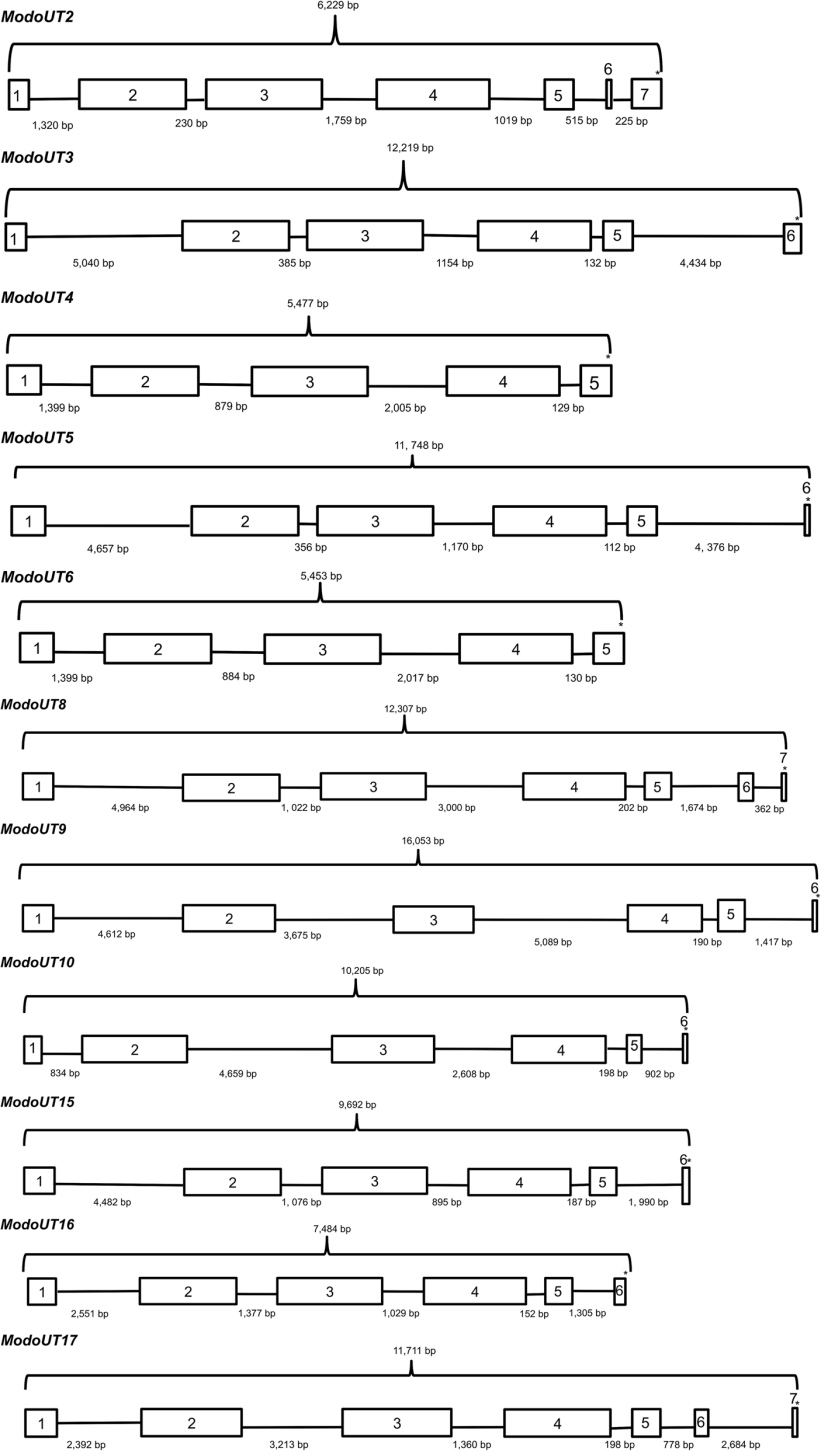
Gene organization and exon composition of *ModoUT2-6, UT8-10, UT15-17* mRNAs. Asterisks indicate relative location of stop codons

To determine the complete structure of representative *ModoUT* genes, a combination of RACE PCR and the sequence data from the Illumina and Roche 454 databases was utilized (not shown). Sequences from all sources were aligned to the opossum whole genome and contigs representing full-length mRNA sequences for the twelve *ModoUT* genes transcribed in the thymus, allowing for the determination of gene boundaries and alternative splice variants (Fig. 2). The gene structure for *ModoUT1* and *11* through *14* were not completed, as transcripts corresponding to these loci were not found in the thymus transcriptome (Fig. 1).

The size range of the full-length *ModoUT* genes ranged from 5,453 bp (*ModoUT6*) to 16,053 bp (*ModoUT9*). *ModoUT2, 4, 6,* and *16* have similar lengths (5,453–7,484 bp) while *ModoUT3, 5, 8, 9, 10*, *15,* and *17* are larger in size from 9,672 bp to 16,053. As is typical of MHC class I loci, the number of exons per locus varied between five and seven (Fig. 2). The variation in gene size does not correlate with phylogenetic relationship based on coding sequence, as highly related groups like *ModoUT3, 4, 5,* and *6* range from 5,453 bp to 12,219 bp [28].

**Fig. 3:**
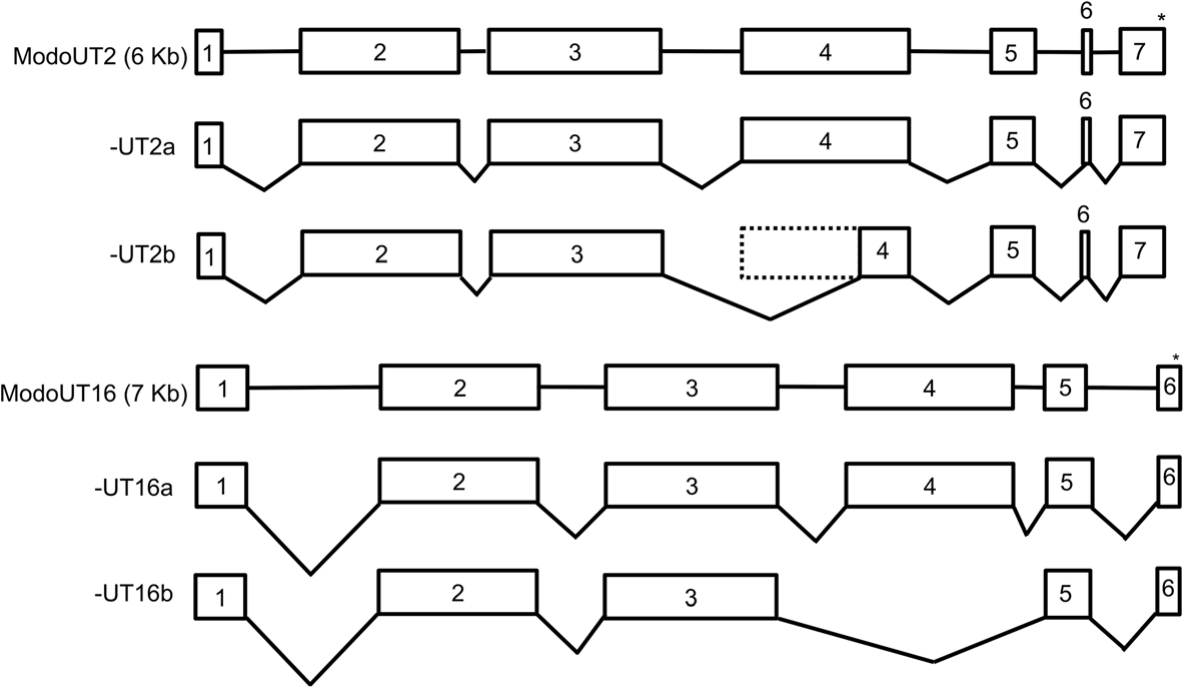
Alternative spliced isoforms of *ModoUT2* and *ModoUT16*. The two are transcribed into alternatively spliced isoforms that differ with the loss or truncation of exon 4. Asterisks indicate relative location of stop codons

Previous work in *M. domestica* has revealed that a majority of the class I loci within the MHC region were transcribed to generate alternative splice variants with open reading frames [21, 24]. Only *ModoUT2* and *UT16* were found with splice variants with open reading frames other than the full length (Fig. 3). The *ModoUT2* splice variant would encode a truncated α3 domain due to using an alternative splice site within exon 4, whereas the *ModoUT16* variant would be translated without an α3 domain due to splicing out exon 4 altogether (Fig. 3).

### Polymorphism and evidence of positive selection

Levels of polymorphism in the *ModoUT* genes were investigated using both captive bred animals from Brazilian stock as well as wild caught Bolivian animals. MHC class I genes typically have the highest level of polymorphism in exons 2 and 3 that encode the α1 and α2 domains, respectively. Genomic DNA corresponding to exon 2, intron 2, and exon 3 was amplified from eight captive bred Brazilian *M. domestica* and 19 Bolivian wild caught animals. At least two independent PCR amplifications were performed for each individual. Total heritable nucleotide alleles, whether the changes are in coding or non-coding sequence of each gene, are presented in Additional file 1: Table S1.

Heterozygosity was common across the UT genes and most frequently seen in the wild-caught Bolivian individuals (Additional file 1: Table S1). Of the captive Brazilian animals, those from Population 2 were homozygous in two-thirds of the *ModoUT* genes analyzed for polymorphism. The high percentage of homozygosity in captive-bred is likely the result of some partial inbreeding [29]. Only four and six animals were found to be homozygous at *ModoUT3* and *ModoUT5*, respectively, while *ModoUT9*, *ModoUT10*, and *ModoUT15* had twenty or more animals that were homozygous. For most of the *ModoUT* genes, approximately half of the animals were heterozygous. Animals collected from the Porvenir region of Bolivia displayed the greatest degree of heterozygosity, with the highest numbers in *ModoUT3*, *5*, *6*, *7*, and *8*.

Translated *ModoUT* sequences revealed substantial amino acid residue conservation and a limited number of protein variants per allele. Allelic variation ranged from two protein sequence variants for *ModoUT9,* to eight for *ModoUT5* due to the presence of non-synonymous substitutions (Additional file 1: Table S1 and Additional file 2: Figure S1). A comparison was made of the number of hydrophobic amino acids in the *ModoUT* translations to other MHC class I molecules (Additional file 3: Figure S2). This included CD1, which has a hydrophobic binding groove. These analyses revealed 41 hydrophobic sites across the ModoUT α1 and α2 domains combined. This is nearly double the number of sites found in classical MHC class I from both eutherians and marsupials where there are typically around 21. The numbers of hydrophobic sites in *ModoUT* genes were comparable to those of CD1 molecules from both eutherian and marsupial mammals, as well as for chicken MHC class I molecule, YF-1 [30].

**Fig. 4:**
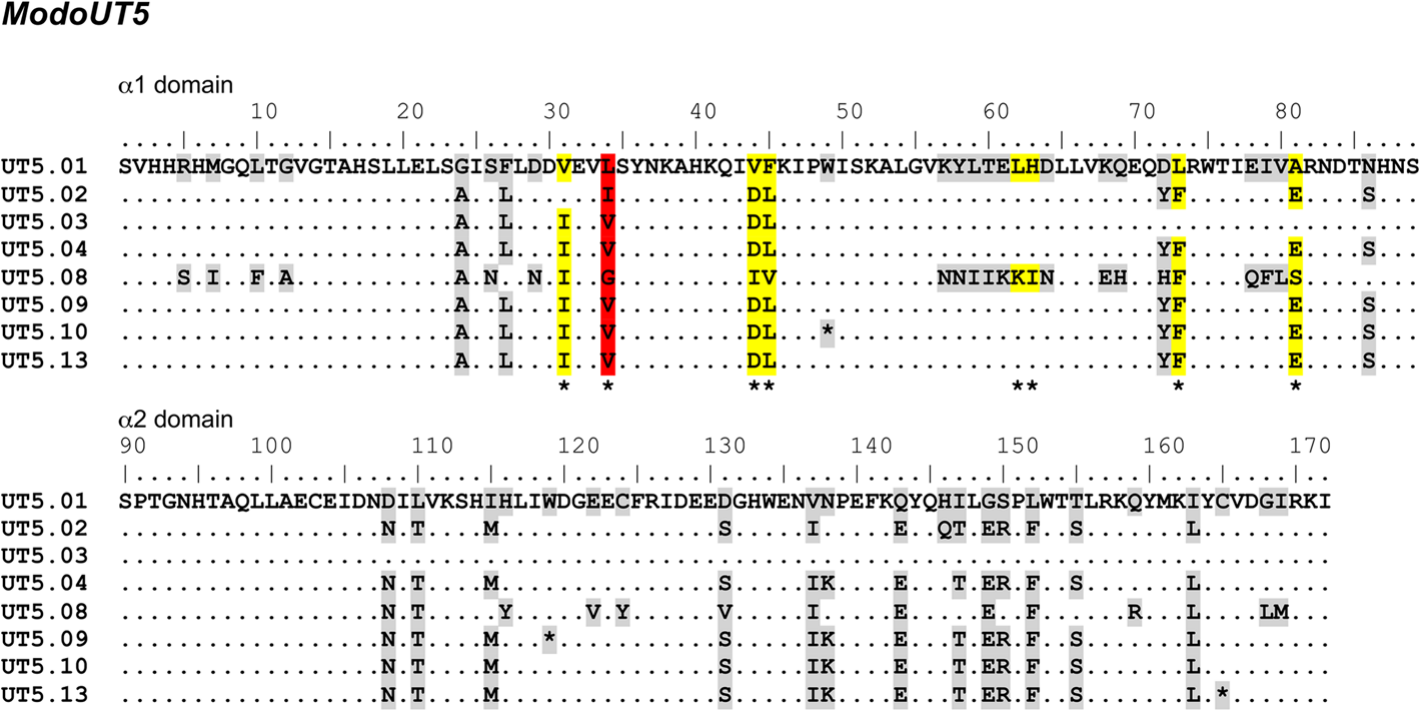
Sites of positive selection in the α1 and α2 domains of *ModoUT5.* *Gray shading* represents sites of polymorphism that are not significant, * represent sites of positive selection, *yellow shading* represents probability of positive selection at specific site at *P* > 95 % (* significant), while *red shading* is significant for probability of selection at *P* > 99 % (** highly significant). Of 52 sites of non-synonymous substitutions across both the α1 and α2 domains, only one was highly probable to be significant, and seven were significant

Positive selection was inferred for the α1 and α2 domains of all *ModoUT* alleles individually with the Pairwise Analysis of Maximum Likelihood method [31]. Only ModoUT5 had eight sites under positive selection, and these were all in the α1 domain (Fig. 4). The predicted ModoUT6 amino acid sequence had a single site with evidence of being under selection. This was in the α2 domain at position 138 where a valine is substituted by a threonine residue (not shown). All other *ModoUT* loci revealed no evidence of positive selection on the protein structure they encoded.

**Fig. 5:**
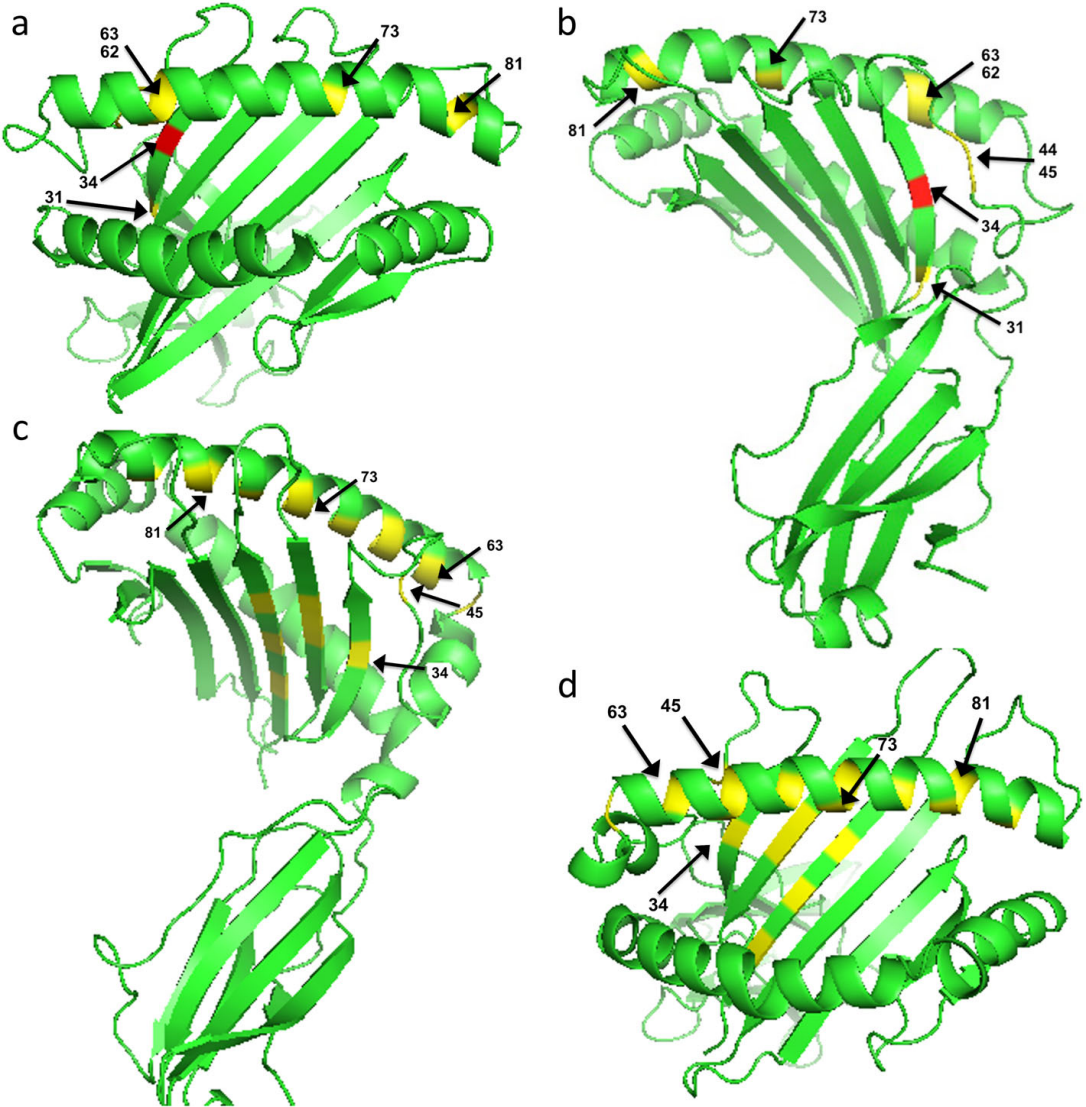
Predicted structural model of *ModoUT5* binding groove identifying the sites of positive selection in comparison to *ModoUA1*. **a** and **b** *Top* and *side views* of predicted structural model of *ModoUT5.* *Red* and *yellow shading* represent sites under positive selection, with *red* significant at *p* < 0.005 and *yellow* at *p* < 0.05. **c** and **d** *Top* and *side views* of predicted structural model of *ModoUA1,* with *yellow shading* representing sites under positive selection

Since the predicted ModoUT5 protein had multiple sites with evidence of positive selection we wished to map where these sites were likely to be in the extra-cellular domains. A structural model of the ModoUT5 was made and the sites under positive selection were highlighted (Fig. 5). All the sites under selection are in the α1 domain (Figs. 4 and 5). The site under the strongest selection (*P* > 99 %; residue 34 in red in the figure) is located in a beta strand that contributes to the peptide-binding region. Four of the more weakly selected sites (*P* > 95 %; yellow in the figure) are located on the alpha helix of the α1 domain.

### Expression of ModoUT8 in thymic but not peripheral αβ T cells

**Fig. 6:**
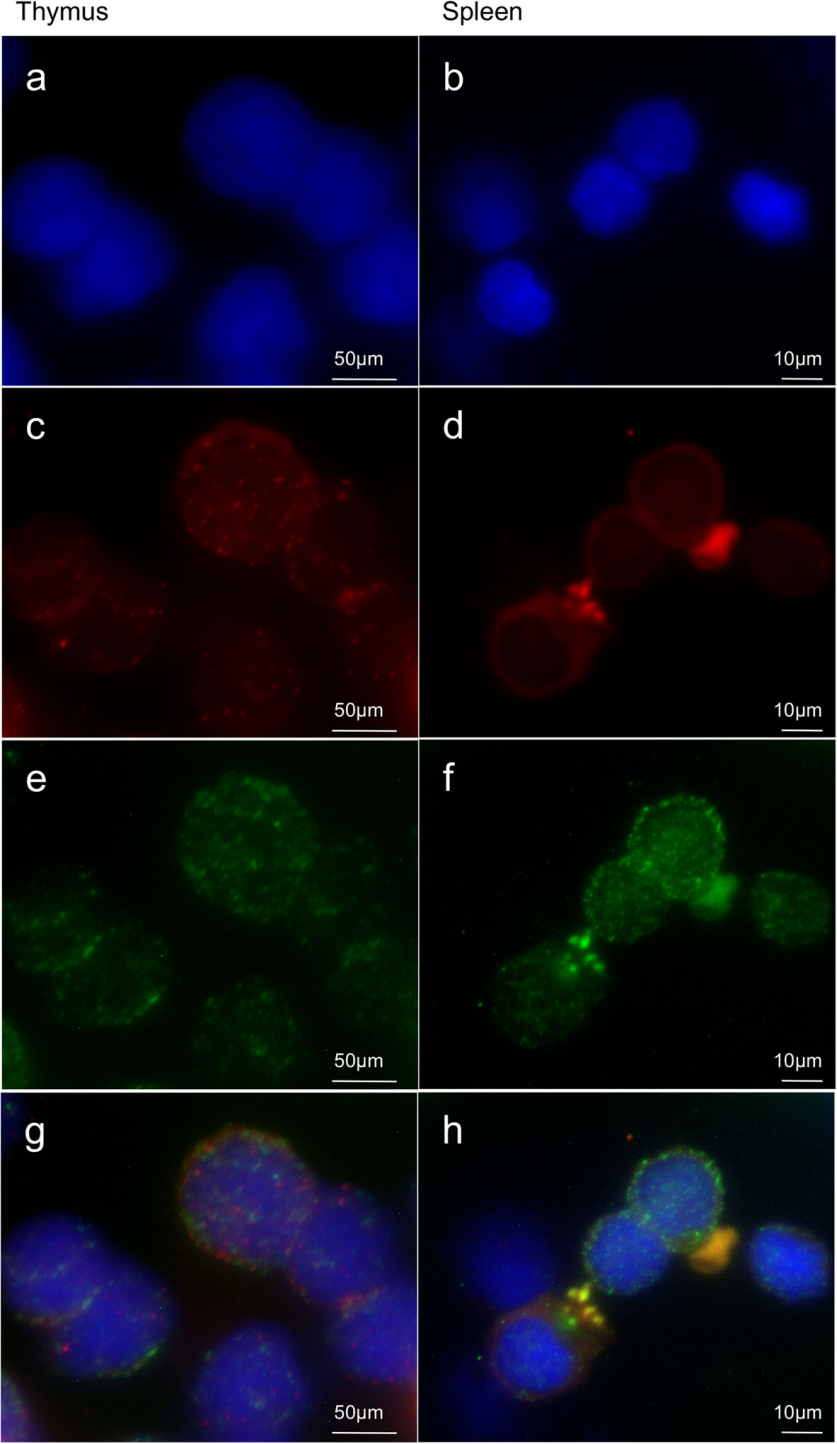
Fluorescent *in-situ* hybridizations of *ModoUT8* with TCRα or TCRβ in the spleen and thymus. **a** and **b** DAPI nuclear staining in the spleen and thymus allows visualization of lymphocytes. **c** and **d** *ModoUT8* probes hybridizes to the thymus but not the spleen. *Red points* indicate evidence of transcription in the cell. Large areas of fluorescence are background and associated with binding of probes to dead cells. **e** and **f** TCRβ probes bind to cells in both the spleen and thymus. **g** and **h** Merged images of DAPI, *ModoUT8,* and TCRB give no evidence of *ModoUT8* transcription in the spleen, while *ModoUT8* co-localized with TCRβ in the thymus

*ModoUT8* was chosen as a model *UT* for further analysis as it was the first *ModoUT* uncovered, it demonstrates a limited transcription pattern like most opossum *UT* genes, it has clear orthologues in other marsupial species, and has the most generic structural similarity of the *ModoUT* molecules [28]. Cell specific transcription of *ModoUT8* was investigated using fluorescence *in situ* hybridization (FISH). Opossum thymus and spleen were chosen as positive and negative tissues, respectively, for *UT8* transcripts (Fig. 1). *ModoUT8* and TCRβ mRNA were imaged simultaneously, allowing the visualization of the location of each of the *ModoUT8* or TCR mRNA molecules within a cell. In the thymus, *ModoUT8* coslocalized with both TCRβ (Fig. 6) and TCRα (not shown), demonstrating that it is transcribed in αβ thymocytes. No other thymus cell type was found to be positive for *ModoUT8* transcripts. Consistent with the transcriptome databases (Fig. 1) and RT-PCR experiments (Additional file 4: Figure S3), *ModoUT8* transcripts were not detected in mature, splenic αβ T cells (Fig. 6).

## Discussion

An unexpected result to emerge from the analyses of marsupial and monotreme genomes was the presence of immune related genes that are absent from eutherian genomes. One example is the *UT* family of MHC class I related genes [28]. Another was a non-conventional TCR locus, *TCRμ* [32]. Both *UT* and *TCRμ* genes are found in marsupial and monotreme genomes, consistent with their presence in the last common ancestor of all living mammals [28, 33–35]. Neither of these gene families have been found in any eutherian mammal or non-mammalian genome to date, consistent with their being unique to mammals but lost in the eutherians [28, 33]. The consequences of the *UT* family or *TCRμ* having been lost in the eutherian lineage is a matter of speculation and can only be understood when their function in the species where they are found is determined. Towards this end, this study was convened to further characterize the *UT* gene family in a model marsupial.

The opossum *UT* gene family can be generally described as having limited tissue expression, low levels of polymorphism, and little evidence of positive selection in the extracellular domains. *ModoUT5* is an exception in that it had several sites under selection in the exon encoding the α1 domain. These characteristics are usually indicative of MHC class I related molecules that have non-peptide presenting (non-classical) roles. The genes that encode the *ModoUT* molecules do not have the ubiquitous pattern of transcription like the polymorphic *ModoUA1* locus in *M. domestica. ModoUA1,* along with its nearest related genes *ModoUA3* and *UA4,* is likely involved in peptide presentation based on its similarity to classical MHC class I loci [27].

Thirteen of the *ModoUT* genes are transcribed in three or less tissue types, indicative of a restricted transcription pattern. The majority of *ModoUT* genes are transcribed in the thymus and skin. It is noteworthy that two of the most broadly transcribed *ModoUT* genes*, ModoUT12* and *13*, with six or more sites of transcription, are not transcribed in the thymus. This may indicate that ModoUT12 and 13 are playing a different role than the UT molecules that are found in the thymus and skin. From this observation, these genes may encode molecules with more restricted roles than those with a broader distribution of transcription.

All *ModoUT* loci encode molecules predicted to have hydrophobic residues in the groove created by the α1 and α2 domains [28]. They are also predicted to form a structure most resembling the chicken B21 MHC class I molecule that binds peptides promiscuously [36]. The hydrophobic residues as well as the possibility of non-specific peptide binding due to structure suggest they may be able to present small peptides within the relatively closed binding groove, or they bind hydrophobic antigens such as lipids. A more specific role for *ModoUT* in the thymus may be for T cell selection and maturation. Alternatively, *ModoUT* genes may also just be ectopically expressed in the thymus for negative selection against self-molecules and not serve in T cell selection.

The role of ModoUT in skin is intriguing. The skin serves as a barrier against many pathogens [37]. Close examination of the epithelial tissues other than skin did not find a correlation between epithelium and *ModoUT* transcription. Rather, it is skin specific and may indicate UT molecules playing a role in innate immunity in this particular barrier.

Minimal polymorphism and little evidence positive selection on the *ModoUT* genes follows a similar pattern as other *M. domestica* non-classical MHC class I genes. The lack of polymorphism is consistent with *ModoUT* molecules likely having a function other than peptide presentation.

Noteworthy is the unique transcription pattern of the *ModoUT8* gene*. ModoUT8* transcription is not detected in the spleen by either RNA sequencing or FISH, but its transcripts do co-localize with *TCRα* and *TCRβ* transcripts in thymocytes. This is consistent with mature, peripheral αβ T cells not using *ModoUT8*, however it likely does play a role in developing αβ T cells in the thymus. These results are consistent with it playing a role in T cell maturation. What that role is is not clear. Transcription of the *ModoUT8* gene in the thymus was restricted to cells co-expressing *TCRα* and *TCRβ* transcripts and, therefore, not in epithelial cells. Therefore *ModoUT8’s* role does not appear to be through selection by expression on cortical or medullary epithelial cells. Rather it may serve as a receptor or co-receptor used by developing marsupial αβ T cells.

## Conclusion

Further characterization of the *UT* genes in the opossum supports that some, such as *ModoUT8*, are likely involved in the immune system. Given the patterns of tissue specific transcription and overall lack of polymorphism their roles are likely to be non-classical in nature. The characterization of the *UT* genes in *M. domestica* contributes new insights into genes that are being uncovered using novel methods, but also provides greater knowledge into the evolution and development of marsupials and their immune system and how they differ from eutherian mammals. Future work on *ModoUT* genes and their products will allow for the discovery of the immune and/or non-immune roles they play in marsupials and monotremes.

## Methods

### Compliance with ethical standards

All procedures using live animals were conducted under the approval of the Institutional Animal Care and Use Committee of the University of New Mexico (Protocol Number 13-100920-MCC). No live surgery was performed.

### Tissue transcription of ModoUT

*ModoUT* gene transcription was examined using the Illumina generated transcriptome data from of 19 adult *M. domestica* tissues, publically available at OpossumBase (http://opossumbase.org). Tissues included kidney, liver, lung, spleen, colon, skin (ear pinna), intestine, stomach, diaphragm, heart, skeletal muscle, tongue, pancreas, thyroid, brain, eye, adipose, testes, and ovary. In addition, a thymus transcriptome was generated using thymuses pooled from 6 week-old *M. domestica.* Total RNA was isolated from the thymuses using Trizol extraction protocol. The mRNA was then isolated and purified using the RNA Purelink Mini Kit (ThermoFisher Scientific, Grand Island, NY) and OnColumn DNase I (Sigma Aldrich, St. Louis, MO). A cDNA library was then generated using the GS FLX Titanium Rapid Library Preparation Kit (Roche 454 Life Sciences, Indianapolis, IN). The emPCR amplification of the cDNA library was done using the GS FLX Titanium LV emPCR Lib-L Kit (Roche 454 Life Sciences, Indianapolis, IN), and pyrosequencing was then performed on the Roche 454 GS FLX+ system using the GS FLX Titanium Sequencing XLR70 Kit (Roche 454 Life Sciences, Indianapolis, IN). A total of 543,464 reads were generated, with a mean length of 377 base pairs. The transcriptome was assembled using the GS De Novo Assembler (Roche 454 Sequencing, Indianapolis, IN), resulting in a total of 143,595 reads of contigs or singletons with an average length of 1,026 base pairs for contigs. Sequences obtained from these databases are included in the Genbank accession numbers KP125495 through KP125507.

BLAST searches of each of the databases were performed using the predicted nucleotide sequence of exons 2, 3 and 4 that encode the regions corresponding to the α1, α2, and α 3 domains from all *ModoUT* genes identified previously [24]. Sequences in the assembled RNA databases that were over 180 bp and 98 % or greater nucleotide sequence identity to a known *UT* gene were scored as transcripts of that gene. In order to verify the accuracy of the transcription sites, any transcripts of *ModoUT* genes uncovered using BLAST search were aligned to all the previously identified predicted sequences. Alignments were then assembled into a phylogeny using MEGA 5.0 and maximum-likelihood tree was generated for basic identification of transcripts to a specific *ModoUT* in order to avoid incorrect sites of transcription due to high sequence identity between the *ModoUT* genes [38]. Polymerase chain reactions (PCRs) were done on thymus, spleen, lung, liver, and kidney tissues in *ModoUT8* to confirm same transcription patterns (data not shown).

### Isolation of full-length coding sequences

Two methods were used to isolate full-length coding sequences of transcribed *ModoUT* loci: Rapid Amplification of cDNA Ends (RACE) PCR using thymus complementary DNA (cDNA) and assembly using available transcriptome sequence databases. The latter included OpossumBase and the opossum thymus 454 transcriptome. This approach allowed for assembly of a *ModoUT* gene in the absence of obtaining the full 3’ end via RACE PCR, or to assemble without PCR amplification and sequencing. Full-length coding sequences for *ModoUT4, 5, 6,* and *8* were isolated entirely using the RACE method, while *ModoUT9* and *10* were obtained using a combination of sequences generated by RACE and from the transcriptome databases. RACE PCR were performed using the SMARTer RACE cDNA Amplification Kit (Clontech, Mountain View, CA) with Advantage HF-2 high-fidelity Taq polymerase (Clontech, Mountain View, CA) following the manufacturer’s recommended protocol. PCR parameters for all primers were 94 °C for 30 s, followed by 72 °C for 3 min, repeated 5 times, then 94 °C for 30 s, 70 °C for 30 s, and 72 °C for 3 min repeated 5 times, followed by 94 °C for 30 s, 68 °C for 30 s, and 72 °C for 3 min, repeated 25 times. If no PCR products were visible on an agarose gel, a secondary PCR amplification was done by an additional 5 cycles at 94 °C for 30 s, 68 °C for 30 s, and 72 °C for 3 min. To generate full-length cDNA coding sequences for *ModoUT4* and *8* by RACE, primers were designed to amplify overlapping RACE products. To isolate the 5’ end of cDNA primers were designed based on the exon 3 sequence predicted previously [24]. Similarly, to amplify the 3’ cDNA ends, primers were designed based on predicted exon 2 sequence [28]. It was not possible to design separate 5’ and 3’ primers for *ModoUT5* and *6* that were within the parameters of the amplification kit, so the same sequence in predicted the exons 2 or 3 was used (Table 1). The PCRs were performed on thymus cDNA made from total RNA extracted from a 6-week-old male *M. domestica*. The 5’ and 3’ RACE PCR was performed in a single step for *ModoUT4*, *5*, *9,* and *10* with specific primers (Table 1), or as a nested PCR for *ModoUT6* and *ModoUT8* using additional nested specific primers. PCR products were cloned, and sequenced using BigDye Terminator v3.1 Cycle Sequencing Kit (Invitrogen, Grand Island, NY). Any sequences obtained that did not span the full length of the 3’ end were supplemented using 454 transcripts. The nucleotide sequences from the 5’ and 3’ ends were then aligned against the MonDom5 opossum genome assembly to identify the exons present in each sequence [GenBank; AAFR03000000]. The MonDom5 genome is a well annotated and complete and has been assembled into chromosomes [19].

**Table 1:** List of gene specific primers sets used for PCR and cDNA for RACE

Gene	Exon 2 F	Exon 2 R	Intron 2 F	Intron 2 R	Exon 3 F	Exon 3 R	Length	Overlap
ModoUT2	TGCTCTCCAGCTCTCCACA					ATCTTCAGAAATGTTGGGCACT	689 bp	
ModoUT3	ACCACAGCCATATAGGTCAGTTC					TCAAGTCAACACAATAGAGCTTCA	824 bp 812 bp,	
ModoUT4	CCTTCCTATAATCTAGTCCACCACA		CAATCATGGGGGTTAGAAACAT	TCCCCAAATTCTTAGTGCAGTT		TCCAACAATTTTCTTCATCAAGTC	742 bp	207 bp
ModoUT5	CCTATAATTCAGTCCACCACAGG					CTCACCATTATCCCTTATGCTTG	855 bp 837 bp,	
ModoUT6	CCTTCCTATAATTCAGTCCACCA		GTCCCATGCTCTTTTTCAGTTC	TCTCATGTTATGCCATGTGTCC		TTCCTTAAGTTTGAATACCCAATG	779 bp 753 bp,	244 bp
ModoUT7	CACACTATAACTTCATGTAGTGTTTCC		GCTGCTTGCAGGAGTAAAAGAT	GACTTTCAGAGTTTGCCAAAGG		CCTATAATTCAGTCCACCACAGG	682 bp 816 bp,	150 bp
ModoUT8	GTTCTGCAGCTCACCACAAAC		CTCATCCACCAGTGTGTTGTTC	GGGTAGGAGCTTCATGTCAGTC		CTTGCATGTAGTGCTTCCTCTG	738 bp 286 bp,	96 bp
ModoUT9	CCCATATATGACCTGCTTTCCT	GTGGAGGTGGAAATAAGTGAGG			TTAGCCTGGCCATAATACTTCC	GGGAAATATGACTTGTACCTCCTG	450 bp 346 bp,	
ModoUT10	CCCCTACCTTTCTGTCATTCAG	GGACAAGCGCTTTTTGAGTATG			TTCATCTCCAATTCTGATTCCA	GATTGGTTAGAGCCTGTGCTTT	354 bp 833 bp,	
ModoUT15	GCTTTCACTCCTGTCTCTCAGC		ACCTCGAAGGTCATCTAGTCCA	TGTCCTTTGAAACCTAGGCAAT		TCATGCTTGAATGCTGTAGAAC	876 bp 229 bp,	138 bp
ModoUT17	CTTTTCTGTCCTTTAGCCCATC	CCCCTCTCTTATCATTCTTTACC			CCAGAGAACCATACAGTCCAGAT	TGTTTGACTGTAGGACTTTCCTC	210 bp	
	5’ RACE R	5’ RACE R nested	3’ RACE F	3’RACE F nested	overlap			
ModoUT4	TTCATGTTCTCCCAGTGCCCAACC		GAAACCACACAGCCCAGCTCCAG		89 bp			
ModoUT5	TGTGCCAACTCCAGTCAACTGTCCC		GGGACAGTTGACTGGAGTTGGCACA		0 bp			
ModoUT6	AGACTGGTCCAGAGGGGACTCTC		CTGGAGAGTCCCCTCTGGACCAGTCT		0 bp 363 bp,			
ModoUT8	GTGGTCCACAGGTTACTTGTCA	TGGATTTCATTTCCTAGCTGGT	GGAAGCTCCCTCTTGGAATACT	TCATAGAGGACACTTGGCAGAA	119 bp			

Full-length coding sequences for *ModoUT2, 3, 15, 16,* and *17* were determined using only transcripts from tissues transcribed from OpossumBase or from thymus 454. Transcribed *ModoUT* loci were assembled and aligned against the opossum genome in Sequencher 5.0 (Gene Codes, Ann Arbor, MI). In some instances, full-length genes could be found from a single transcript from the transcriptome, while others required multiple transcripts to generate a contig that covered all of the exons. Full-length RNAs encoding the class I α chains *of ModoUT2, 3, 4, 5, 6, 8, 9, 10*, *15*, *16,* and *17* including alternative mRNA splice variants were determined. Their sequences have been deposited into Genbank as accession numbers KP125495 through KP125507.

### Polymorphism analyses

Polymorphism was determined for exons 2 and 3 of those *ModoUT* genes that are transcribed in the thymus. These represent the majority of loci and those for which full-length gene structure could be determined. Genomic DNA was isolated from *M. domestica* liver or spleen tissue that was provided by the Southwest Foundation for Biomedical Research (San Antonio, TX) and the Museum of Southwestern Biology, University of New Mexico [21, 24]. DNA was extracted using a standard phenol/chloroform extraction protocol. The Southwest Foundation for Biomedical Research tissues were from captive-bred animals of Brazilian populations 1 and 2 [29]. Tissues from the Museum of Southwestern Biology were from wild-caught *M. domesti*ca collected from five different sites in Bolivia. These samples and their origins have been published previously [17, 20]. PCR products using primers listed in Table 1 were amplified using the Advantage HF 2 PCR kit (Clontech, Mountain View, CA), with the following parameters for all primers: 94 °C for 1 min, 33 cycles of 94 °C 30 s and 62 °C for 4 min, followed by a 7 min extension at 68 °C.

For loci where intron 2 was less than 400 bp (*ModoUT2, UT3, and UT5*), exon 2, intron 2, and exon 3 were amplified as a single product with primers placed at the start of exon 2 and end of exon 3. For loci where intron 2 was greater was greater than 800 bp (*ModoUT4, UT6, UT7, UT8, and UT15*), exon 2, intron 2, and exon 3 were amplified as two overlapping fragments with primers nested within intron 2. Three loci (*ModoUT9, UT10*, and *UT17*) had intron 2 greater than 3Kb and exons 2 and 3 were amplified separately.

PCR products were cloned for sequencing using the TOPO TA cloning kit (Life Technologies, Grand Island, NY). A minimum of 5 clones per individual per locus were isolated and sequenced using the illustra TempliPhi kit (GE Life Sciences, Pittsburgh, PA) and BigDye Terminator v3.1 Cycle Sequencing Kit (Life Technologies, Grand Island, NY), respectively. Sequences were analyzed and edited using Sequencher 5.0 (Gene Codes, Ann Arbor, MI) and BioEdit [39]. [GenBank: *ModoUT2*, KP174147-KP174156; *ModoUT3*, KP221763-KP221785; *ModoUT4*, KP174157-KP174166; *ModoUT5*, KP221786-KP221799; *ModoUT6*, KP174167-KP174180; *ModoUT7*, KP221744-KP221762; *ModoUT8*, KP174181-KP174197; *ModoUT9*, KP245840, KP245841, KP245858-KP245860; *ModoUT10*, KP245842-KP245847; *ModoUT15*, KP174198-KP174203; and *ModoUT17*, KP245848-KP245857].

Evidence of selection on the ModoUT loci was determined using a maximum-likelihood estimation of the dN/dS ratio using the CODEML module of the Phylogenetic Analysis by Maximum Likelihood (PAML) package [31]. The regions encoding the α1 and α2 domains of all *ModoUT* alleles obtained from the polymorphism study were analyzed for evidence of positive selection by looking at non-synonymous/synonymous substitution ratios (*d*N/*d*S). This was done using an initial Maximum Likelihood phylogenetic tree of sequences that was then analyzed with three site-specific models, M0 (one ratio), M1a (nearly neutral), and M2a (positive selection) in CODEML. Defined parameters include codon frequency mode of F3X4, no molecular clock, equal amino acid distance, and estimated ω and κ. The model M0 was used to estimate tree branch lengths, M1a looked at restricted sites with ω (*d*N/*d*S) ≤ 1, and model M2a included sites with ω > 1. A Bayes Empirical Bayes analysis was performed on each model to infer the location of the codons under selection and the ω values for each of these sites [40]. Values of ω < 1, = 1, and >1 were considered indicative of purifying selection, neutral evolution and positive selection, respectively. Sites with a value of ω >1 across the Bayes Empirical Bayes analysis were inferred to be under positive selection. The probability of site undergoing selection at specific sites was measured at *P* > 95 % (* significant) and *P* > 99 % (** highly significant).

A predicted structural model of *ModoUT5* was generated using the Protein Homology/analogY Recognition Engine (PHYRE2) using a template from the chicken YF7.1 [41]. The PDB was visualized using the PyMOL Molecular Graphics System, Version 1.2 (Schrödinger, LLC).

### Fluorescent In-situ Hybridization (FISH)

Thymus and spleen tissue from six-week old opossums were collected for cell suspensions. The tissues were manually separated into single cell suspensions that were washed and resuspended in RPMI 1640 media with HEPES (Sigma-Aldrich, St. Louis, MO). Erythrocytes were removed by Ficoll gradient centrifugation. Lymphocytes were resuspended in 1X PBS (Sigma-Aldrich, St. Louis, MO) and counted on a hemocytometer. Cells at a concentration of 2x10^6^/ml were deposited on non-coated Shandon Cytoslides using a Cytospin (Thermo-Fisher Scientific, Waltham, MA) and used immediately or stored frozen at −80 °C. Custom Stellaris® FISH Probes were designed against the constant regions of TCRs and UTRs for T cell identification and full-length sequence of *ModoUT8* by utilizing the Stellaris® RNA FISH Probe Designer (Biosearch Technologies, Inc., Petaluma, CA) available online at www.biosearchtech.com/stellarisdesigner (Version 2). Probes contained 48 singly labeled oligonucleotide probes, each 20 nucleotides in length that were designed from the full-length sequences or constant regions. *ModoUT8* was hybridized with the Quasar® 570 fluorophore and TCRα or TCRβ hybridized with the Quasar® 670 fluorophore for the Stellaris RNA FISH Probe set (Biosearch Technologies, Inc.), following the manufacturer’s instructions for frozen tissue available online at www.biosearchtech.com/stellarisprotocols. Slides were then stained using wash buffer consisting of 5 ng/mL of DAPI dilactate (Sigma-Aldrich, St. Louis, MO) and mounted using Vectrashield® Mounting Medium (Vector Labs, Burlingame, CA). Slides were imaged on a Nikon Ti Eclipse inverted fluorescent microscope using the 100x oil immersion lens. Z-stacks images of each fluorophores were merged together and edited using NIS-Elements Imaging Software (Nikon Instruments, Melville, NY).

## Additional files

Additional file 1: Table S1. The numbers of alleles varies between 2 and 23, but overall low levels of polymorphism were found. (PDF 62 kb)
Additional file 2: Figure S1. Yellow shading represents conserved hydrophobic regions, and red circles show three sites of selection in the *ModoUT5* alleles that are found in the hydrophobic regions. (TIFF 8149 kb)
Additional file 3: Figure S2. Comparison of identity across *ModoUT* alleles in the α2 domain. Yellow shading represents conserved hydrophobic regions. (TIFF 5534 kb)
Additional file 4: Figure S3. Agarose gel containing RT-PCR products using *ModoUT8* specific primers revealing presence of transcripts in thymus RNA but not spleen or liver. (PDF 657 kb)

## Abbreviations

cDNA: Complementary DNA
FISH: Fluorescent in-situ hybridization
*M. domestica*: *Monodelphis domestica*
MHC: Major Histocompatibility Complex
PAML: Phylogenetic Analysis by Maximum Likelihood
PCR: Polymerase chain reaction
RACE: Rapid amplification of cDNA ends
TCR: T cell receptor
UTR: Untranslated region
