# Supplementary material for: The UT family of MHC class I loci unique to non-eutherian mammals has limited polymorphism and tissue specific patterns of expression in the opossum
Source: BMC Immunol. 2016 Nov 8;17:43. doi: 10.1186/s12865-016-0181-9 (PMC5101759; doi:10.1186/s12865-016-0181-9)
Supplement: Additional file 1: Table S1. — The numbers of alleles varies between 2 and 23, but overall low levels of polymorphism were found. (PDF 62 kb) [file 12865_2016_181_MOESM1_ESM.pdf]

Table S1: Genotypes of *M. domestica* used in this study.

Animal[1]	Source[2]	UT2	UT3	UT4	UT5	UT6	UT7	UT8	UT9	UT10	UT15	UT17
C7585	1	01/01	01/02	01/01	01/01	01/01	01/02	01/02	01/01	01/01	01/01	01/01
C6076	1	01/02	03/04	01/02	01/02	01/01	03/03	03/04	02/02	01/02	01/02	01/05
D0214	1	01/01	03/05	01/01	01/01	01/01	01/01	03/03	01/01	01/01	01/01	01/01
D3888	1	03/04	03/06	03/04	03/04	02/03	n.d.	05/06	02/02	03/05	02/02	02/06
D4368	1	02/02	03/07	05/05	01/02	01/02	04/05	05/06	02/02	02/03	03/04	01/11*
D2718	1	01/01	03/08	01/01	01/05	01/01	01/01	03/03	01/01	01/01	01/01	02/02
D2920	2	01/02	03/03	02/02	01/04	04/04	06/06	04/04	02/02	03/03	02/02	02/06
D5468	2	02/02	03/09	05/05	01/04	05/05	07/07	06/06	02/02	03/03	05/05	03/03
23169	Brecha Tres	01/05	10/11	06/07	06/06	06/06	08/08	07/08	03/03	02/02	06/06	04/07
21696	Rio Limon	06/06	n.d.	07/07	06/07	07/08	09/10	04/09	03/03	04/04	06/06	04/04
12653	Porvenir	06/06	10/10	07/07	06/07	06/07	09/11	08/08	03/03	02/02	06/06	04/04
12664	Porvenir	06/06	10/12	07/08	06/06	08/09	08/09	10/11	03/03	02/02	06/06	04/04
23170	Brecha Tres	06/06	12/13	07/07	08/09	08/08	12/12	08/08	03/03	02/02	06/06	04/04
12663	Porvenir	06/07	10/12	08/09	06/09	07/08	10/13	08/09	03/03	02/02	06/06	04/08
12668	Porvenir	06/08	12/14	07/09	08/10	06/09	14/15	08/12	03/03	02/02	06/06	04/04
12669	Porvenir	06/09	n.d.	n.d.	06/07	06/06	15/16	10/13	03/03	01/02	06/06	08/09
221692	S.R. de A.	08/10	15/15	06/07	06/08	10/11	17/17	08/08	03/03	02/02	06/06	04/08
12670	Porvenir	06/08	11/16	07/10	06/06	10/10	11/13	08/09	03/03	01/02	06/06	04/08
12538	Tita	06/08	17/17	07/07	06/06	07/07	09/09	08/08	03/03	01/02	06/06	04/08
12350	Santiago de Chiquitos	n.d.	15/18	07/07	08/11	08/12	11/18	14/15	03/03	02/02	06/06	04/10
12571	Porvenir	n.d.	10/19	07/07	06/12	06/06	15/19	07/16	03/03	02/02	06/06	04/04
12555	Porvenir	06/06	10/12	07/07	06/12	09/13	12/17	08/16	03/03	02/02	06/06	04/04
12622	Porvenir	07/07	10/20	07/09	06/09	06/08	15/20	08/09	03/03	02/02	06/06	04/04
12580	Porvenir	06/06	12/21	07/07	06/13	06/08	11/15	16/16	03/03	02/02	06/06	04/04
12578	Porvenir	n.d.	18/22	07/09	06/13	06/09	14/21	08/17	03/03	02/02	06/06	04/04
12579	Porvenir	06/08	15/21	07/07	06/09	08/09	09/15	10/16	03/03	02/02	06/06	04/04
12623	Porvenir	n.d.	15/23	07/09	06/14	09/14	17/22	08/08	03/03	01/02	06/06	04/04

[1] Identifier numbers for individual *M. domestica* of Brazilian origin from the Southwest Foundation for Biomedical Research (SFBR) breeding colony or for wild-caught Bolivian animals in the frozen tissue collections at the Division of Genomic Resources, Museum of Southwestern Biology (MSB), University of New Mexico, respectively.
[2]Source refers to the Brazilian SFBR breeding population (Population 1 or 2) from which animal is descended from or the site of local collection of wild Bolivian *M. domestica* from the MSB collection.
n.d. indicates animals whose genotype was not determined at the indicated loci, * an unconfirmed but likely allele.
