# Supplementary figures and images for: The UT family of MHC class I loci unique to non-eutherian mammals has limited polymorphism and tissue specific patterns of expression in the opossum
Source: BMC Immunol. 2016 Nov 8;17:43. doi: 10.1186/s12865-016-0181-9 (PMC5101759; doi:10.1186/s12865-016-0181-9)

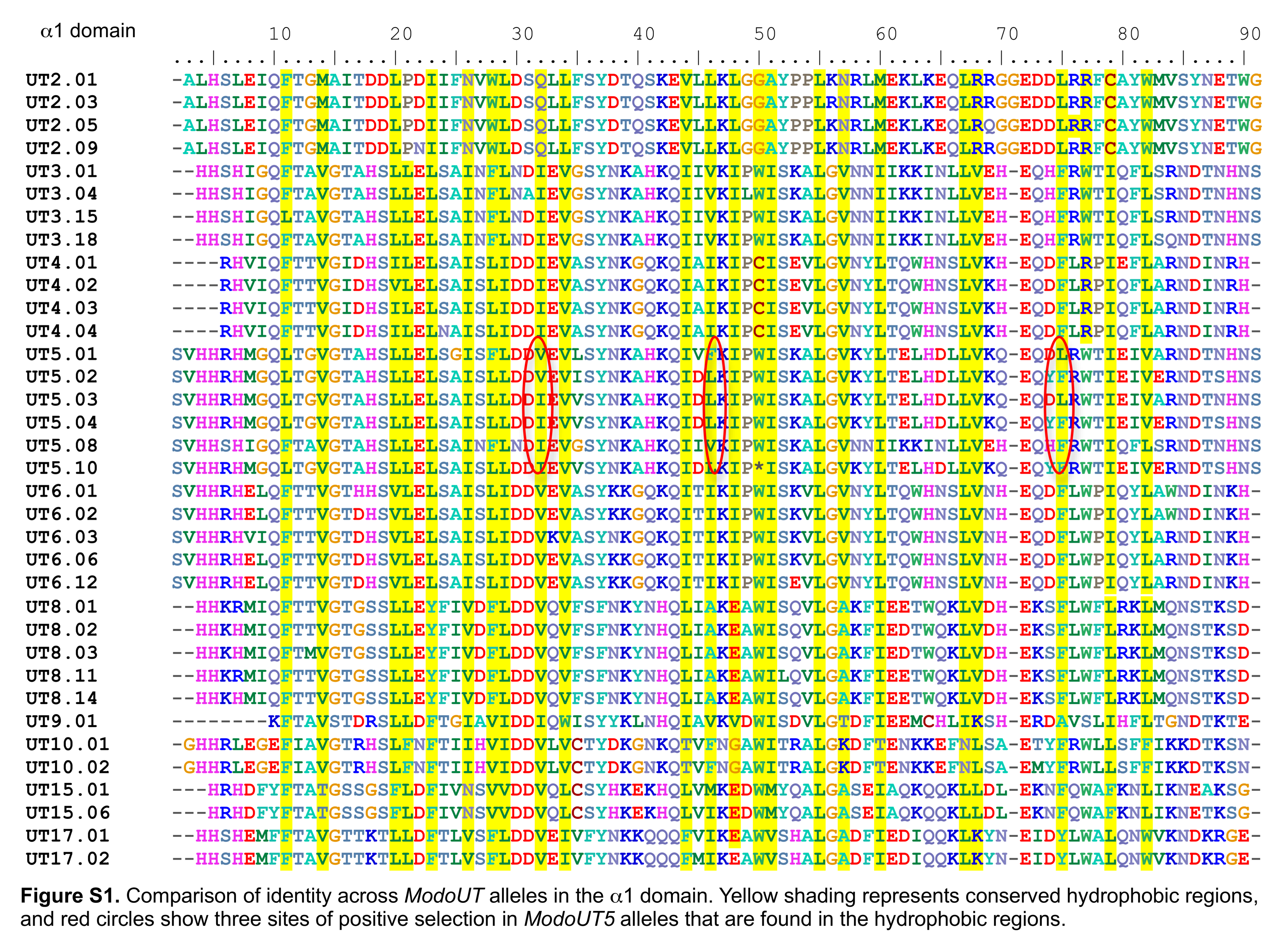

Supplement: Additional file 2: Figure S1. — Yellow shading represents conserved hydrophobic regions, and red circles show three sites of selection in the ModoUT5 alleles that are found in the hydrophobic regions. (TIFF 8149 kb) [file 12865_2016_181_MOESM2_ESM.tiff]

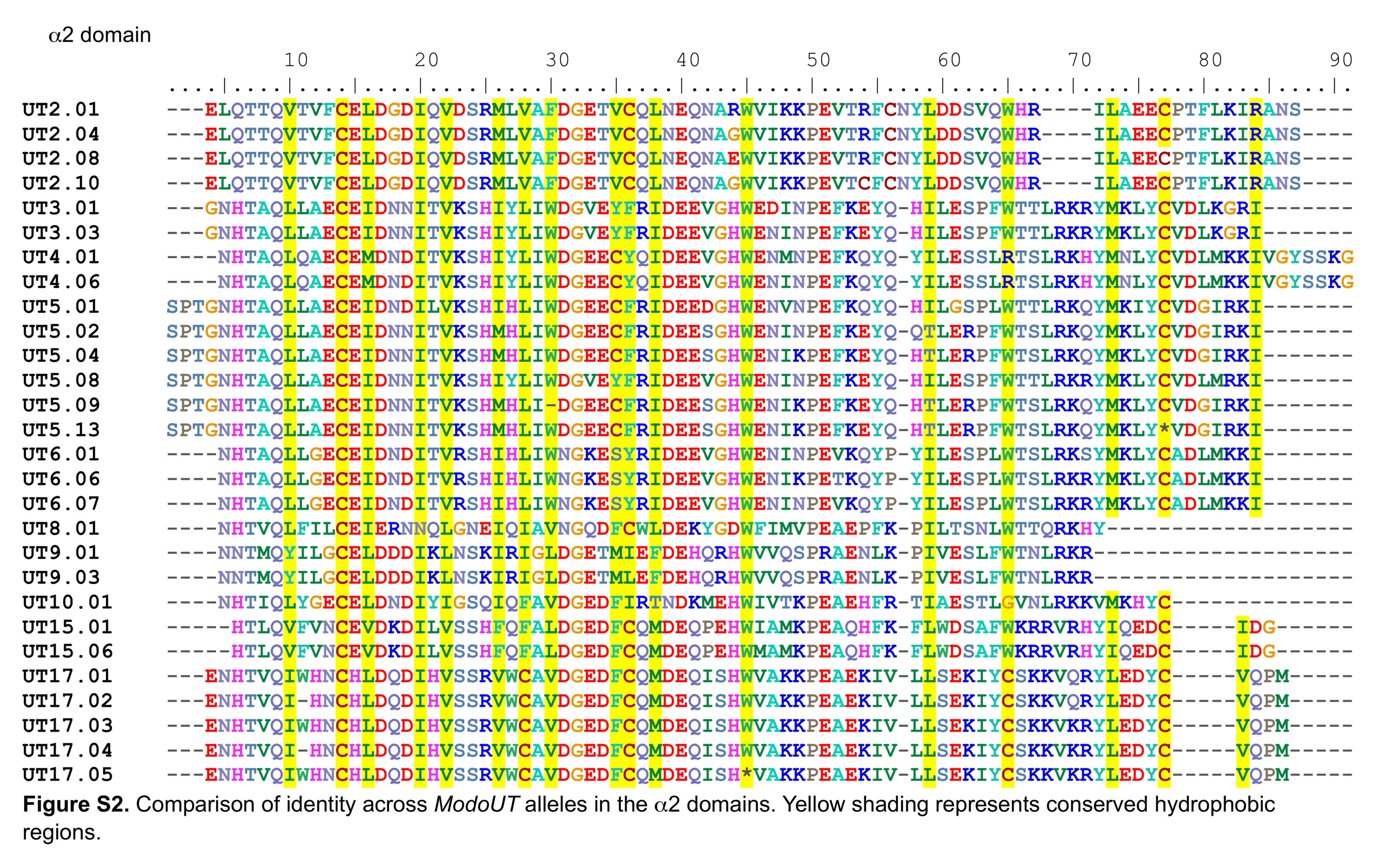

Supplement: Additional file 3: Figure S2. — Comparison of identity across ModoUT alleles in the α2 domain. Yellow shading represents conserved hydrophobic regions. (TIFF 5534 kb) [file 12865_2016_181_MOESM3_ESM.tiff]

Size marker

Thymus RNA

Spleen RNA

Liver RNA

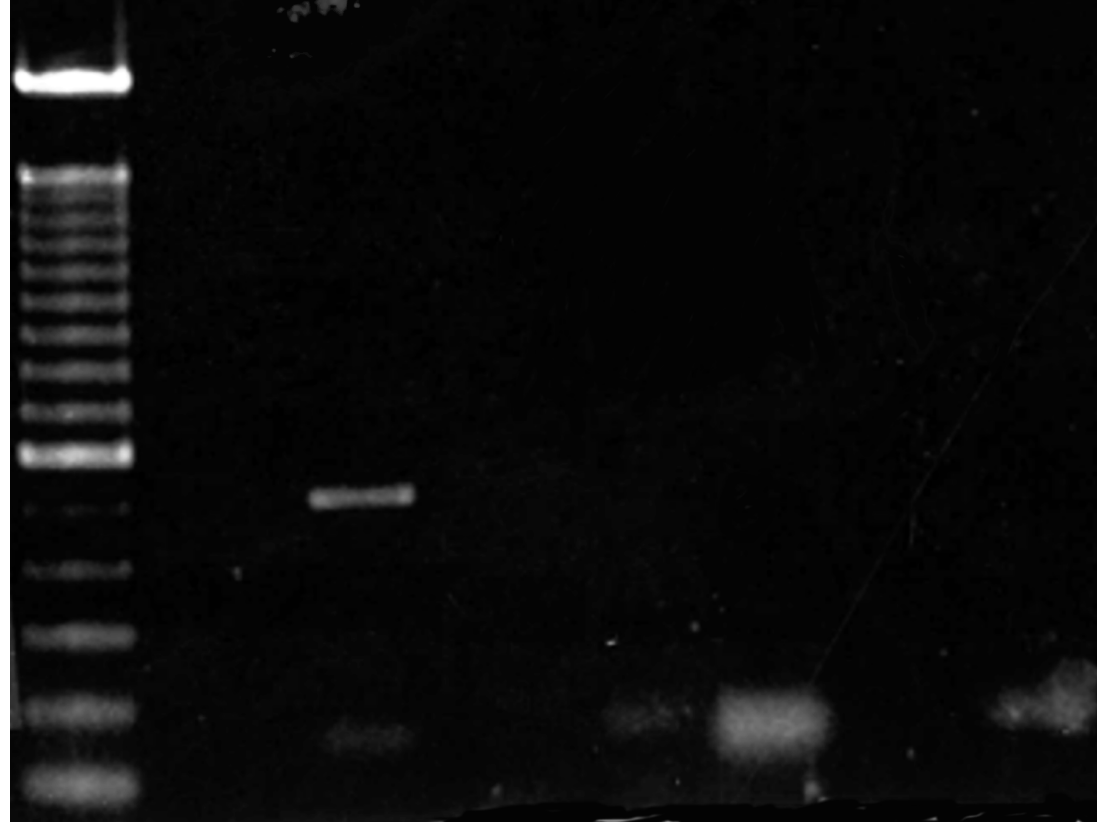

Supplement: Additional file 4: Figure S3. — Agarose gel containing RT-PCR products using ModoUT8 specific primers revealing presence of transcripts in thymus RNA but not spleen or liver. (PDF 657 kb) [file 12865_2016_181_MOESM4_ESM.pdf]
